# Slit2/Robo4 Signaling Modulates HIV-1 gp120-Induced Lymphatic Hyperpermeability

**DOI:** 10.1371/journal.ppat.1002461

**Published:** 2012-01-05

**Authors:** Xuefeng Zhang, Jinlong Yu, Paula M. Kuzontkoski, Weiquan Zhu, Dean Y. Li, Jerome E. Groopman

**Affiliations:** 1 Division of Experimental Medicine, Beth Israel Deaconess Medical Center, Harvard Medical School, Boston, Massachusetts, United States of America; 2 Department of Medicine and Molecular Medicine Program, University of Utah, Salt Lake City, Utah, United States of America; Emory University, United States of America

## Abstract

Dissemination of HIV in the host involves transit of the virus and virus-infected cells across the lymphatic endothelium. HIV may alter lymphatic endothelial permeability to foster dissemination, but the mechanism is largely unexplored. Using a primary human lymphatic endothelial cell model, we found that HIV-1 envelope protein gp120 induced lymphatic hyperpermeability by disturbing the normal function of Robo4, a novel regulator of endothelial permeability. HIV-1 gp120 induced fibronectin expression and integrin α_5_β_1_ phosphorylation, which led to the complexing of these three proteins, and their subsequent interaction with Robo4 through its fibronectin type III repeats. Moreover, pretreatment with an active N-terminus fragment of Slit2, a Robo4 agonist, protected lymphatic endothelial cells from HIV-1 gp120-induced hyperpermeability by inhibiting c-Src kinase activation. Our results indicate that targeting Slit2/Robo4 signaling may protect the integrity of the lymphatic barrier and limit the dissemination of HIV in the host.

## Author Summary

The most common route of HIV transmission is through unprotected sexual contact. By this route, HIV first infects cells in the mucous membranes of the mouth, vagina or rectum. From the mucosa, virus and virus-infected cells move through lymphatic endothelial channels to draining lymph nodes where they infect various cells, including their major target cells, CD4^+^ T lymphocytes. The virus and infected cells then transmigrate through the lymphatic barrier, enter the blood stream, and spread throughout the body. We found that HIV-1 gp120 compromises the lymphatic endothelial barrier by inducing hyperpermeability. We hypothesize that an impaired barrier may facilitate the dissemination of HIV. Likewise maintaining a “normal” barrier may help slow the dispersal of HIV, thereby protecting the body from HIV spread and progression after initial mucosal exposure. We demonstrated that in lymphatic endothelium the interactions among Robo4, Slit2, fibronectin and α_5_β_1_ integrin modulate the effect of HIV-1 gp120 on lymphatic permeability. Moreover, we found that Slit2 inhibits the complexing of Robo4 with fibronectin and protects cells from gp120-induced hyperpermeability. These data suggest that by interacting with Robo4, Slit2 may help maintain the integrity of the lymphatic barrier, thereby interfering with the dissemination of HIV beyond the draining lymph nodes.

## Introduction

HIV becomes established at mucosal sites by infecting dendritic cells, CD4^+^ T lymphocytes and macrophages in the lamina propria after its entry. From there, virus and infected cells disseminate via lymphatic endothelial channels to the draining lymph nodes, and subsequently pass into the bloodstream [1]–[4]. An impaired lymphatic barrier may accelerate HIV dissemination.

Generally, endothelial cells do not express CD4, the major receptor of HIV, but express varying levels of CXCR4 [5] and CCR5 [6], the co-receptors of HIV, depending on the tissue of origin [7]. While HIV can infect endothelial cells, its biological importance in the pathogenesis of AIDS is unclear [8]–[10]. The HIV-1 envelope glycoprotein gp120 and the HIV transactivator of transcription (Tat) may contribute to HIV-associated vasculopathy. HIV-1 gp120 induces apoptosis in endothelial cells [11], [12] and Tat stimulates angiogenesis [13], [14], which is often concomitant with hyperpermeability. Current knowledge of the effects of HIV-associated hyperpermeability are limited to disrupting the integrity of vascular structures and/or enhancing inflammatory reactions. However, these phenomena are characteristic of many infectious diseases [15] and do not explain the unique biology of HIV. In addition, while a pivotal role for the lymphatic system in the pathogenesis of HIV/AIDS has been suggested [16], the pathobiology of HIV interaction with lymphatic endothelium has not been extensively characterized.

The Slit2/Robo4 (Roundabout 4) signaling pathway is a recently identified regulator of endothelial permeability [17]. The Slit/Robo family members were originally discovered as axon guidance molecules that mediate repulsive signaling mechanisms in the central nervous system [18]–[20]. Recent studies from animal models strongly implicate a central role for Slit/Robo in vascular biology [17], [21]. For example, Robo4 knockdown zebrafish embryos have vascular sprouting defects [22], and Robo4 knockout mice display abnormal vascular hyperpermeability [17]. Moreover, Slit2/Robo4 interactions can maintain the integrity of the vascular network and its barrier function by inhibiting cytokine-mediated vasculogenesis and enhanced permeability [17], [23], and the Slit2-Robo4-paxillin-GIT1 network inhibits neovascularization and vascular leakage [24].

Slit2 belongs to a family of three glycosylated extracellular proteins containing at least four different motifs and sharing cognate Robo receptors (Robo1-4) [25], [26]. Slits are secreted by midline glial cells and other tissues [19], [27], [28], and can be processed by proteolytic cleavage to yield a shorter C-terminus fragment of unknown function and a longer, active N-terminus fragment that agonizes the Robos [29], [30]. Robo4 is predominantly expressed in endothelial cells, including embryonic endothelium and tumor vascular endothelium, and shows significant structural differences from the other Robos [26], [31]. Robo4 has only two immunoglobulin (Ig) domains and two fibronectin type III domains in the extracellular region, whereas the other Robos have five and three, respectively [32], [33]. The Robo4 cytoplasmic domain also differs from the other family members, e.g. while Robo1 has four conserved motifs in this region, Robo4 retains only two [26]. Structure-effect studies have revealed that the Slits bind via their N-terminal leucine-rich repeat domain to the Robos, and that the first Ig domain of the Robos is highly conserved and important for Slit binding [34]–[36].

Slit2/Robo4 signaling activates Rho GTPases in endothelial cells, but the precise mechanism by which they interact with each other remains controversial [37]–[39]. There are two prevailing hypotheses for their interaction. One posits that Slit2 activates Robo4 and initiates a signaling cascade [17], [21]. Alternatively, Slit2 may interact with Robo1, and then transactivate Robo4 [39], [40].

In this study, we explored if and how HIV-1 gp120 modulates the Slit2/Robo4 signaling pathway in primary human lung lymphatic endothelial cells. We found that HIV-1 gp120 elevated fibronectin levels, activated fibronectin and α_5_β_1_ integrin, and induced a physical association between α_5_β_1_ and Robo4. This complexing of Robo4 resulted in hyperpermeability in a lymphatic cell monolayer; however, pretreatment with Slit2N, an active N-terminal fragment of Slit2, inhibited significantly these HIV-1 gp120-induced effects. We suggest that the Slit2/Robo4 pathway may play a key role in modulating HIV-1 gp120-induced lymphatic hyperpermeability, and its manipulation may be used to inhibit the dissemination of HIV in the host.

## Results

### HIV-1 gp120 Induces Hyperpermeability of a Lymphatic Cell Monolayer

**Figure 1:**
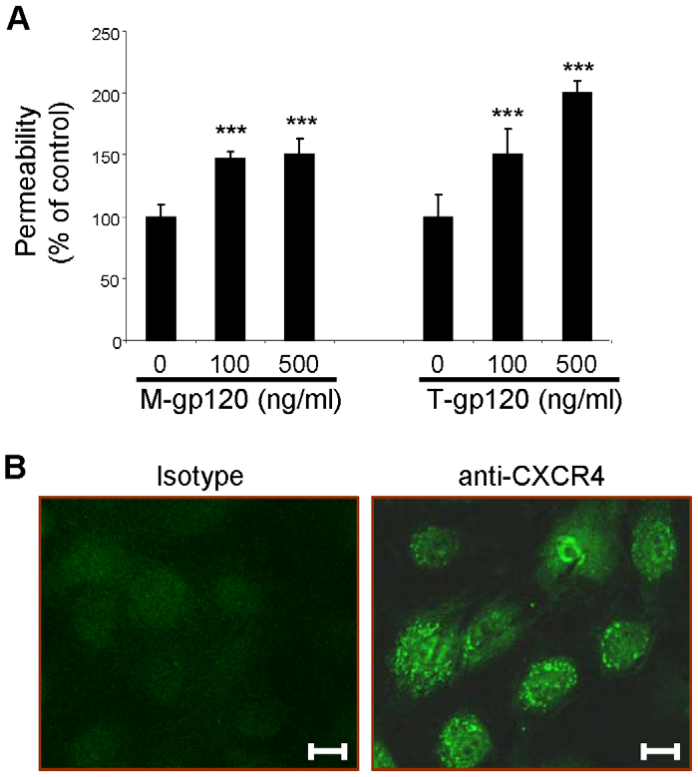
HIV-1 gp120 induces hyperpermeability of a lymphatic cell monolayer. (**A**) Permeability, as determined by the translocation of FITC-conjugated dextran particles through an L-LEC monolayer in transwell chambers. Cells were incubated with M-gp120, T-gp120 or controls at various concentrations for 18 hours. Dextran particles were added, and fluorescence assessed after 5 minutes. Permeability was calculated based on the relative fluorescence of media in lower chambers of HIV-1 gp120-treated cells vs. the controls (“0”). Data indicate the mean ± SD of 3 independent experiments. (*** p<0.001). (**B**) Immunofluorescent analysis of CXCR4 expression in L-LECs. Scale bars = 20 µm. Representative images are shown.

The effects of HIV-1 gp120 on vascular endothelium have been well characterized [41]–[43], however, very little is known about how HIV-1 gp120 specifically affects the lymphatic barrier. To address this issue, we studied the effects of HIV-1 gp120 from two different HIV-1 strains (M-gp120 which utilizes the CCR5 co-receptor on target cells, and T-gp120 which utilizes the CXCR4 co-receptor) on lung lymphatic endothelial cells (L-LECs) in an *in vitro*, vascular permeability assay. Permeability was quantified by the translocation of FITC-conjugated Dextran particles through an L-LEC cell monolayer seeded in the top chamber of a transwell plate, into the bottom chamber, after incubation with specified concentrations of M-gp120 or T-gp120. We observed a significant increase in permeability of the lymphatic cell monolayer after treatment with both M-gp120 and T-gp120 (Figure 1A). We then assessed in the L-LECs, the expression of CD4 (the major receptor for HIV-1 gp120 on target cells) and the co-receptors, CCR5 and CXCR4, by immunohistochemistry. While we detected no expression of CD4 or CCR5 in these cells (data not shown), we observed a robust expression of CXCR4 on the cell surface and in the nucleus (Figure 1B). However, inhibiting the effects of CXCR4 with a neutralizing antibody had no effect on the HIV-1 gp120-induced permeability of the monolayer (data not shown). These data suggest that HIV-1 gp120 induces hyperpermeability in an L-LEC monolayer by a mechanism independent of CD4, CCR5 and CXCR4 binding.

### HIV-1 gp120 Modulates the Expression of Fibronectin and Slit2 in L-LECs

**Figure 2:**
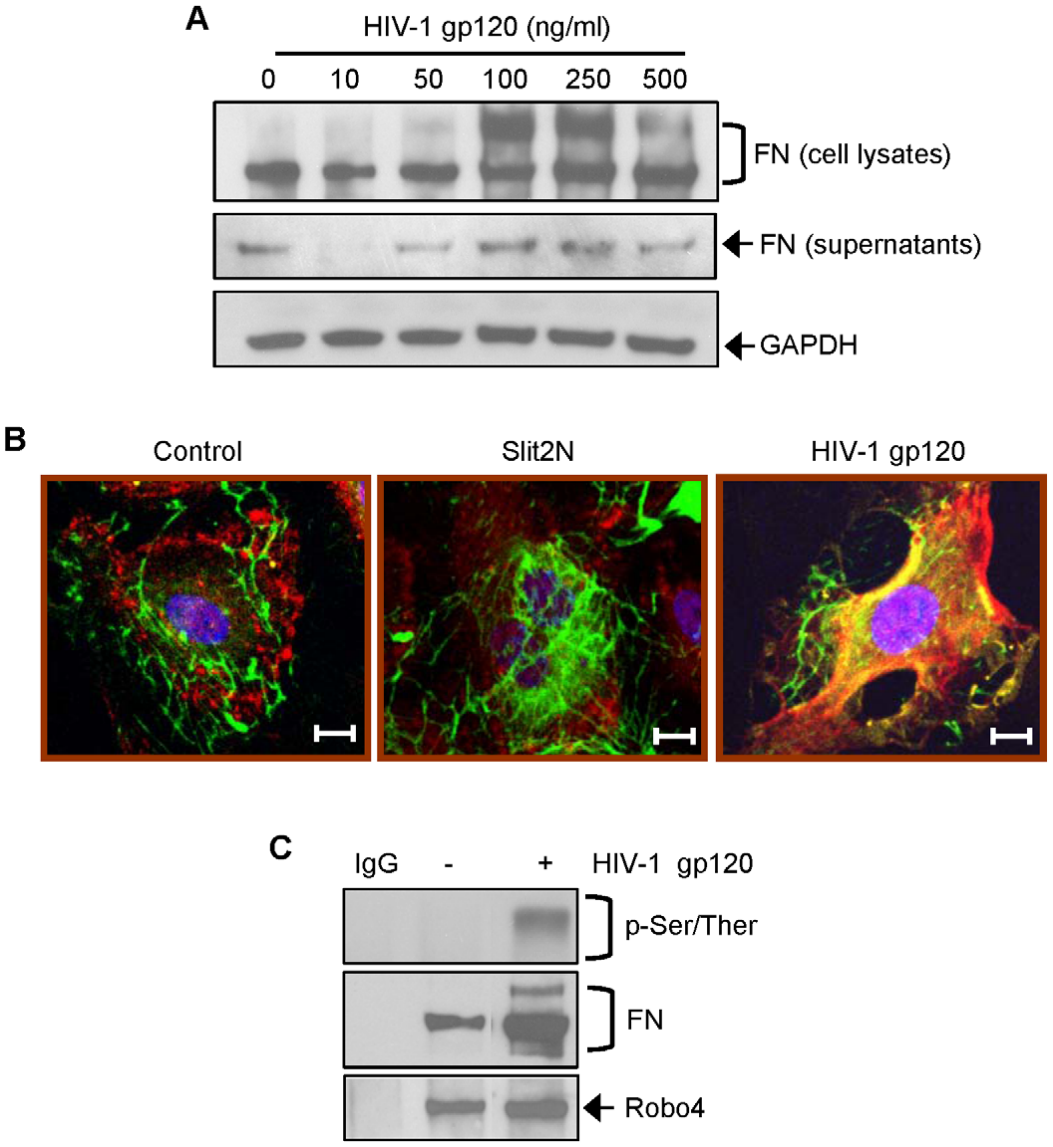
HIV-1 gp120 modulates the expression of fibronectin and Slit2 in L-LECs. (**A**) Representative Western blot analysis of fibronectin (FN) expression in L-LEC cytoplasm and supernatant. Cells were serum-starved for 1 hour and incubated with indicated concentrations of HIV-1 gp120 for 18 hours before harvesting protein. GAPDH used as loading control. (**B**) Robo4 and FN expression in L-LECs by confocal microscopy. L-LECs were cultured in chamber slides and incubated with either HIV-1 gp120 (500 ng/ml), Slit2 (500 ng/ml) or a control for 15 minutes before fixing and staining cells. Red = Robo4; Green = FN; Blue = DAPI. Scale bars = 10 µm. (**C**) Robo4 immunoprecipitation of total and phosphorylated FN (p-Ser/Ther) by Western blot analysis in L-LECs. Cells were incubated with either HIV-1 gp120 (500 ng/ml) or a control for 15 minutes before protein from total cell lysates was collected for Robo4 immunoprecipitation. Membrane was stripped and reprobed for Robo4 expression as a loading control.

Fibronectin is important for maintaining vascular integrity [44] and is involved in lymphangiogenesis [45], [46]. Previous studies showed that HIV-1 gp120 can bind to fibronectin through its heparin-binding domains, and facilitate HIV infection [47]–[49]. Therefore, we assessed fibronectin expression by Western blot analysis in L-LECs and their supernatant after incubation with various concentrations of HIV-1 gp120 (M-gp120 was used in all experiments unless specifically stated otherwise). We observed marked increases of fibronectin (predominantly as a dimer) in cell lysates after treatment with HIV-1 gp120, and less pronounced increases of soluble, monomeric fibronectin in the supernatant (Figure 2A). We interpret our data to indicate that HIV-1 gp120 can enhance fibronectin expression in lung lymphatic endothelial cells. Interestingly, we observed that low concentrations of gp120 (10–50 ng/ml) induced a decrease in FN secretion (vs. untreated) as compared with higher gp120 concentrations (100–500 ng/ml). Few experimental studies focus on gp120 at such low levels, however, we hypothesize that the effects of gp120 at these low concentrations may be an *in vitro* correlate for HIV latent infection *in vivo* and a low viral load, although this has yet to be confirmed.

With the recent discovery that Slit2/Robo4 signaling regulates endothelial permeability [17], [24], and our data that demonstrate HIV-1 gp120-induced hyperpermeability and fibronectin up-regulation in L-LECs, we postulated that fibronectin, Slit2 and Robo4 may be interacting to regulate lymphatic permeability after HIV exposure. By confocal microscopy, we observed the expression and localization of fibronectin and Robo4 in L-LECs with or without treatment with Slit2N or HIV-1 gp120. After stimulation with Slit2N, no co-localization of fibronectin and Robo4 was observed (Figure 2B, middle panel). When the L-LECs were treated with HIV-1 gp120, however, fibronectin and Robo4 displayed strong co-localization (Figure 2B, right panel). These expression patterns and interactions were corroborated by a Robo4 immunoprecipitation assay in which L-LECs, stimulated with HIV-1 gp120, showed fibronectin activation (by serine/threonine phosphorylation, Figure 2C) and a significantly enhanced physical association between fibronectin and Robo4 (Figure 2C).

**Figure 3:**
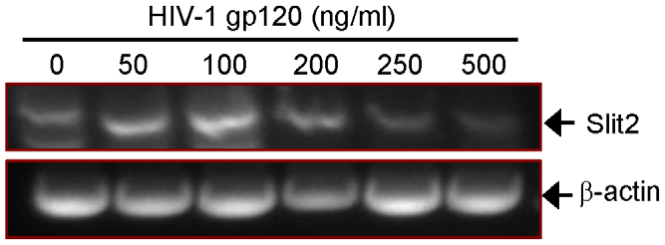
The differential effects of HIV-1 gp120 concentrations on Slit2 expression in L-LECs. Representative RT-PCR analysis (DNA gel) of Slit2 expression in L-LECs after incubation with designated concentrations of HIV-1 gp120 for 18 hours prior to performing RT-PCR. β-actin was amplified as an internal control.

Since Slit2/Robo4 signaling is known to inhibit cytokine-induced vascular permeability [17], [23], we compared Slit2 expression in L-LECs in the presence or absence of HIV-1 gp120, a known inducer of endothelial permeability. Using a semi-quantitative RT-PCR assay, we found that at low concentrations, HIV-1 gp120 enhanced Slit2 expression in L-LECs, while higher concentrations of HIV-1 gp120 inhibited the expression of Slit2 (Figure 3). The inhibition of Slit2 by HIV-1 gp120 at 250 ng/ml and 500 ng/ml is consistent with the characterization of Slit2 as an inhibitor of pathological hyperpermeability [24]. Taken together, these data suggest that fibronectin, Robo4 and Slit2 may cooperate in mediating permeability induced by HIV-1 gp120 in lymphatic endothelium.

### HIV-1 gp120 Induces Hyperpermeability in L-LECs through Activation of α_5_β_1_ Integrin

**Figure 4:**
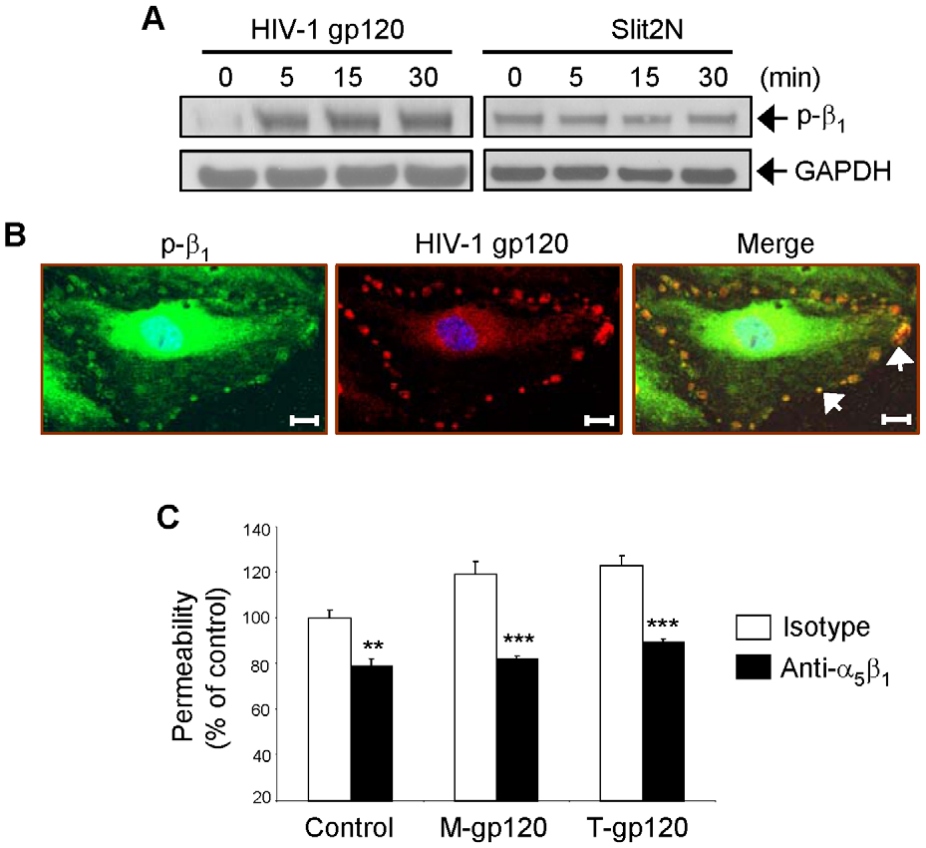
HIV-1 gp120 induces hyperpermeability in L-LECs through activation of α_5_β_1_ integrin. (**A**) Representative Western blot analysis of phosphorylated α_5_β_1_ integrin in L-LECs after 2 hours of serum starvation and subsequent incubation for designated times with HIV-1 gp120 (500 ng/ml) or Slit2N (500 ng/ml). GAPDH used as loading control. (**B**) Phosphorylated α_5_β_1_ integrin and HIV-1 gp120 expression and co-localization in L-LECs by confocal microscopy. L-LECs were cultured in chamber slides and incubated with HIV-1 gp120 (500 ng/ml) for 15 minutes before fixing and staining cells. Scale bars = 10 µm. (**C**) Permeability through an L-LEC monolayer as previously described. L-LEC monolayers were pretreated with a neutralizing anti-integrin β_1_ antibody or a normal IgG control for 2 hours before incubating with M-gp120 or T-gp120 (both 500 ng/ml) for 18 hours. Data indicate the mean ± SD of 3 independent experiments. (**p<0.01; *** p<0.001).

When fibronectin interacts with vascular endothelium it commonly binds to either α_5_β_1_ integrin or α_v_β_3_ integrin on the cell surface. Activation of these integrins dramatically enhances this interaction [50]. Therefore, we examined the expression of these two integrins in L-LECs by Western blot analysis. Since we detected α_5_β_1_, but not α_v_β_3_ in L-LECs (data not shown), we investigated only α_5_β_1_ in subsequent experiments. We treated L-LECs for various times with either HIV-1 gp120 or Slit2N. By Western blotting we measured the levels of β_1_ phosphorylation, a reflection of α_5_β_1_ activation (Figure 4A). We observed no change in α_5_β_1_ activation after Slit2N treatment, however, incubation with HIV-1 gp120 induced significant phosphorylation of β_1_ (Figure 4A). Furthermore, we observed co-localization of HIV-1 gp120 and activated α_5_β_1_ integrin on the L-LEC cell surface by confocal microscopy (Figure 4B, “Merge” panel).

Based on these results, we hypothesized that the physical interaction between HIV-1 gp120 and integrin α_5_β_1_ may play a role in HIV-1 gp120-induced effects. Therefore, we examined the effect of blocking this interaction on lymphatic hyperpermeability. Using the previously described *in vitro* transwell permeability assay, cells were pre-treated with either a neutralizing anti-α_5_β_1_ antibody or an isotype control before incubation with M-gp120 or T-gp120. We observed increased permeability through the L-LEC monolayer after treatment with either of the HIV-1 gp120 isotypes (Figure 4C); pretreatment with the anti-α_5_β_1_ antibody prevented much of the increase in permeability associated with HIV-1 gp120 (Figure 4C). These data indicate that the increased activation of α_5_β_1_ integrin by HIV-1 gp120 and their physical association are required for HIV-1 gp120-induced hyperpermeability of L-LECs.

### Slit2N Inhibits HIV-1 gp120-Induced Lymphatic Hyperpermeability by Blocking the Interaction between α_5_β_1_ and Robo4

**Figure 5:**
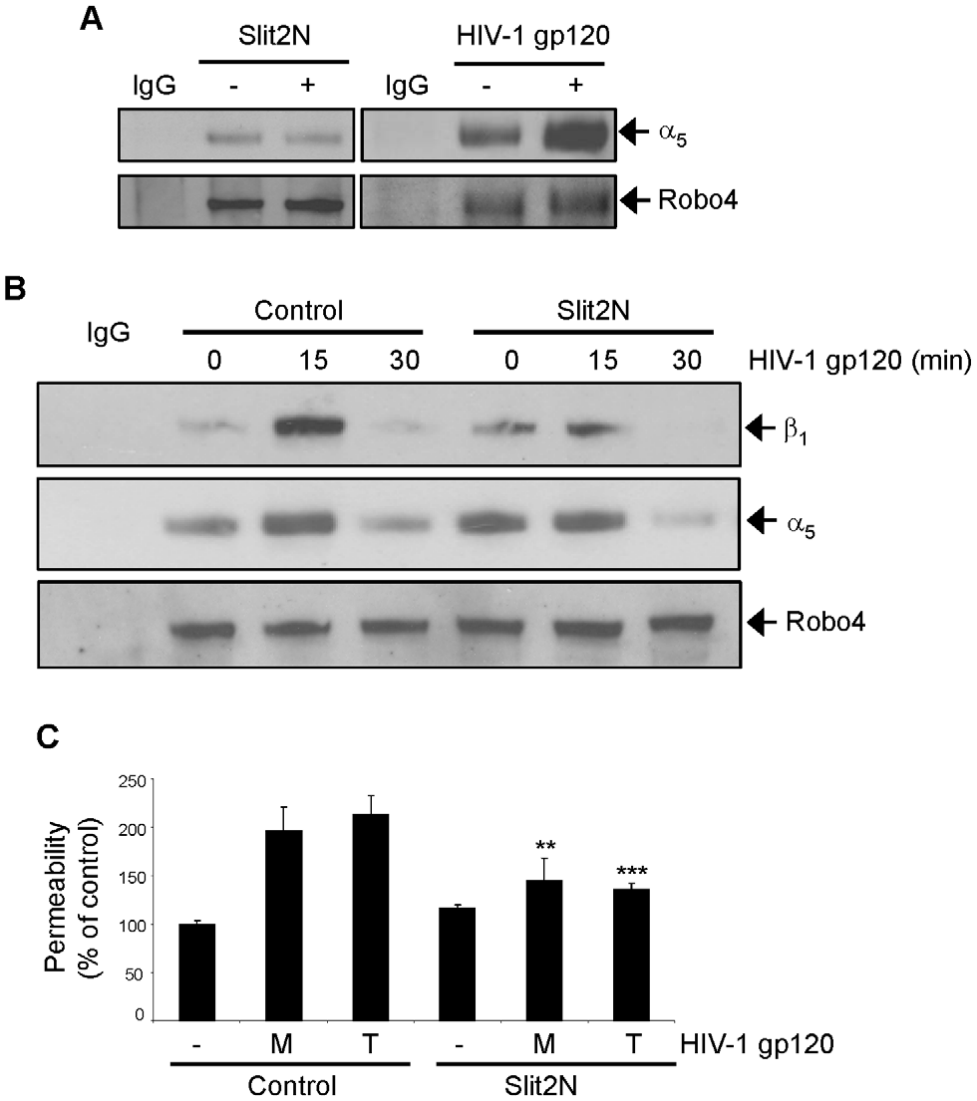
Slit2N inhibits HIV-1 gp120-induced lymphatic hyperpermeability by blocking the interaction between α_5_β_1_ integrin and Robo4. (**A**) Robo4 immunoprecipitation of α_5_β_1_ integrin by Western blot analysis in L-LECs. Cells were incubated with either Slit2N (500 ng/ml), HIV-1 gp120 (500 ng/ml) or their respective controls (“−”) for 15 minutes before protein from total cell lysates was collected for Robo4 immunoprecipitation. Membranes were stripped and reprobed for Robo4 expression as a loading control. (**B**) Robo4 immunoprecipitation of α_5_β_1_ integrin by Western blot analysis in L-LECs. Cells were incubated with either Slit2N (500 ng/ml) or a control for 2 hours and then treated with HIV-1 gp120 (500 ng/ml) for times indicated before the protein from total cell lysates was collected for Robo4 immunoprecipitation. Membrane was stripped and reprobed for Robo4 expression as a loading control. (**C**) Permeability through an L-LEC monolayer as previously described. L-LEC monolayers were pretreated with Slit2N (500 ng/ml) or a control for 2 hours before incubating with M-gp120 or T-gp120 (both 500 ng/ml) for 18 hours. Data indicate the mean ± SD of 3 independent experiments. (**p<0.01; *** p<0.001 for treatment with Slit2N versus vehicle control).

Based on the results from our expression and co-localization studies of Slit2, Robo4, gp120, fibronectin and α_5_β_1_, we sought to investigate further the physical interactions that contribute to HIV-1 gp120-induced hyperpermeability, and to explore the specific effects of Slit2 on these processes. To these ends, we examined the physical interaction of Robo4 and α_5_β_1_ in L-LECs after treatment with Slit2N or HIV-1 gp120 in a Robo4 immunoprecipitation assay. The basal association between Robo4 and α_5_β_1_ integrin was not affected by the differential expression of Slit2N (Figure 5A). However, we observed a significant increase in this physical association after treatment with HIV-1 gp120 (Figure 5A). We then pretreated L-LECs with Slit2N or a negative control before incubating the cells with HIV-1 gp120. While the association between Robo4 and α_5_β_1_ integrin appeared to peak 15 minutes after HIV-1 gp120 incubation (Figure 5B), pretreatment with Slit2N greatly diminished this interaction (Figure 5B). Based on these data, we theorized that Slit2 may antagonize the effects of HIV-1 gp120 on a lymphatic cell monolayer, and therefore, may protect lymphatic endothelium against HIV-1 gp120-induced hyperpermeability. To test this hypothesis we utilized the L-LEC transwell permeability assay previously described. While incubation with M-gp120 and T-gp120 increased L-LEC monolayer permeability (Figure 5C, “Control” bars), the extent of this HIV-1 gp120-induced hyperpermeability was significantly inhibited by pretreatment with Slit2N (Figure 5C, “Slit2N” bars). We interpret these data to indicate that Slit2N significantly inhibits HIV-1 gp120-induced hyperpermeability in lymphatic endothelium by blocking the physical association between Robo4 and α_5_β_1_ integrin.

### Slit2N Inhibits HIV-1 Virus-Induced Lymphatic Monolayer Hyperpermeability

**Figure 6:**
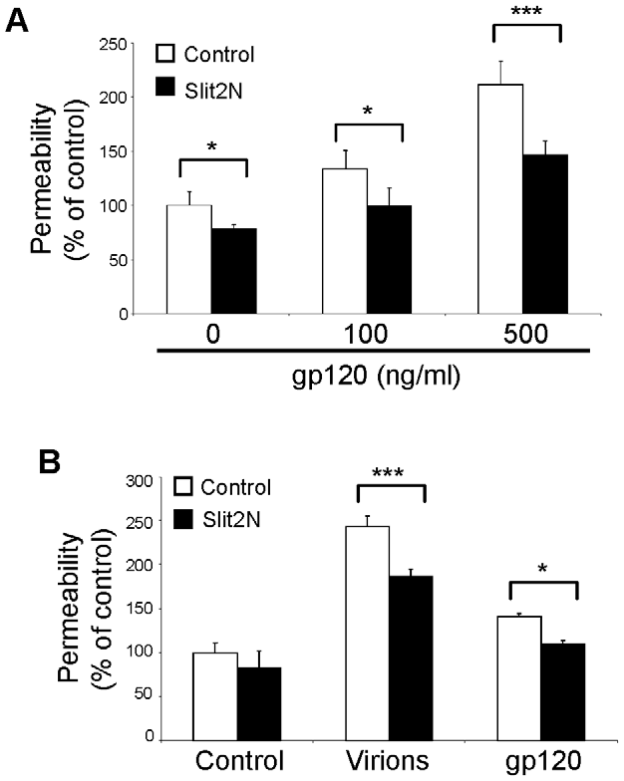
Slit2N attenuates HIV-1 virus-induced lymphatic hyperpermeability. (**A**) Permeability through a dermal lymphatic endothelial cell (D-LEC) monolayer as previously described. D-LEC monolayers were pretreated with Slit2N (500 ng/ml) or a control for 2 hours before incubating with M-gp120 (500 ng/ml) for 18 hours. (**B**) Permeability through an L-LEC monolayer as previously described. L-LEC monolayers were pretreated with Slit2N (500 ng/ml) or a control for 1 hour, followed by incubation with HIV-1 virions (4.0×10^6^ TCID 50/ml) or gp120 (500 ng/ml) for 5 hours. The viral load corresponds to a gp120 concentration of 425 ng/ml. For (**A**) and (**B**), data indicate the mean ± SD of 3 independent experiments. (*p<0.05; *** p<0.001 for treatment with Slit2N versus vehicle control).

To demonstrate that Slit2N and gp120 can induce similar effects in various types of lymphatic endothelium, we repeated the lymphatic permeability assay using primary human dermal lymphatic endothelial cells (D-LECs). Similar to the results using L-LECs, gp120 increased the permeability of D-LEC monolayers in a dose-dependent manner, and pretreatment with Slit2N significantly decreased the gp120-induced hyperpermeability (Figure 6A). To confirm that the changes in permeability were not due to the origin of the gp120, we repeated this experiment with another M-tropic gp120 protein, gp120CM, and observed similar effects (data not shown).

To demonstrate that the effects of the gp120 protein reflect accurately those of intact HIV-1 virions on lymphatic hyperpermeability, we pretreated L-LEC monolayers with Slit2N or a negative control, followed by incubation with HIV-1 virions or gp120. We found that HIV-1 virions significantly increased lymphatic permeability within 5 hours, whereas gp120 induced only a mild increase during the same time period (overnight incubation was needed for full *in vitro* effect of gp120 on permeability) (Figure 6B). Pretreatment with Slit2N significantly inhibited the permeability induced by both the HIV-1 virions and the gp120 protein (Figure 6B). Taken together, our results indicate that intact HIV-1 virions increase lymphatic monolayer permeability, and preincubation with Slit2N can effectively inhibit this increase. These data indicate that HIV-1 virions can induce lymphatic endothelial monolayer permeability similar to that induced by gp120, suggesting that our *in vitro* model of gp120-induced endothelial cell monolayer permeability may reflect the actions of HIV-1 *in vivo*.

### Slit2 and Robo4 Influence HIV-1 gp120-Induced Hyperpermeability in L-LECs by Modulating c-Src Kinase Activation and Signaling

**Figure 7:**
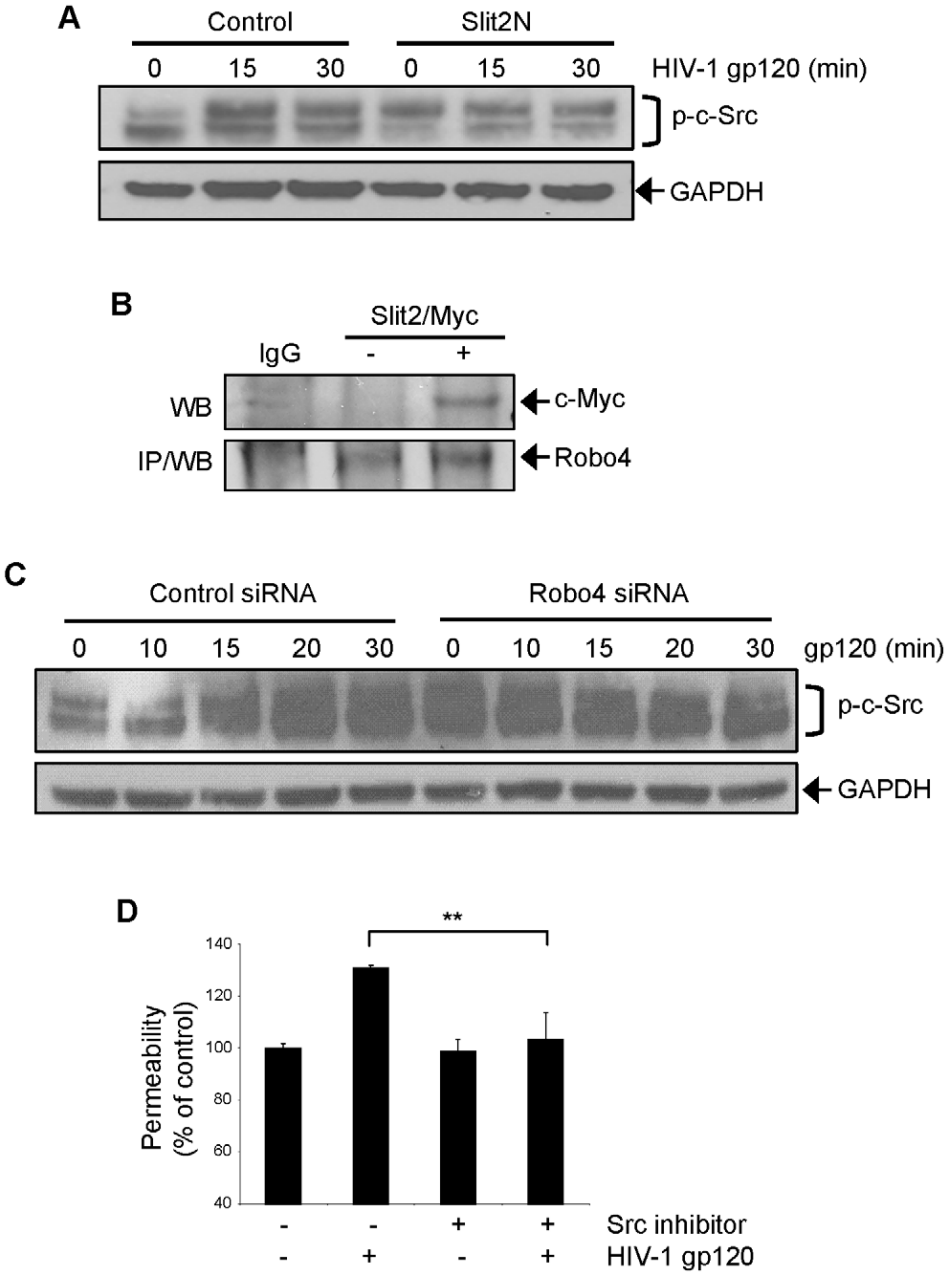
Slit2N and Robo4 influence gp120-induced hyperpermeability in L-LECs by modulating c-Src kinase activation and signaling. (**A**) Representative Western blot analysis of phosphorylated c-Src in L-LECs after preincubation with either Slit2N (500 ng/ml) or a control for 2 hours before treatment with HIV-1 gp120 (500 ng/ml) for times indicated. GAPDH used as loading control. (**B**) Robo4 immunoprecipitation of Myc-tagged Slit2 by Western blot analysis in 293 cells. 293 s were co-transfected with a Robo4 expression plasmid and either a Myc-tagged Slit2 expression plasmid or a vector control. Cells were incubated for 48 hours before the protein from total cell lysates was collected for Robo4 immunoprecipitation. (**C**) Representative Western blot analysis of phosphorylated c-Src in L-LECs after transfection with either Robo4-specific siRNAs or a control siRNA for 48 hours before treatment with HIV-1 gp120 (500 ng/ml) for times indicated. GAPDH used as loading control. (**D**) Permeability through an L-LEC monolayer as previously described. An L-LEC monolayer was pretreated with a Src kinase inhibitor (2 µM) or DMSO for 2 hours before incubating with HIV-1 gp120 (500 ng/ml) or a control for 18 hours. Data indicate the mean ± SD of 3 independent experiments. (**p<0.01 for treatment with the Src kinase inhibitor versus DMSO control).

To elucidate the signaling cascade(s) responsible for HIV-1 gp120-induced hyperpermeability in lymphatic endothelium, we analyzed the effects of HIV-1 gp120, Slit2N and Robo4 by Western blot analysis on Src kinase, a key molecule in the regulation of vascular endothelial permeability [51], [52]. We found that preincubation of L-LECs with Slit2N significantly inhibited HIV-1 gp120-induced phosphorylation of c-Src (Figure 7A), indicating inhibition of the Src signaling pathway. We theorized that the modulation of Src kinase signaling by Slit2N and Robo4 may be the result of a physical complexing between the two proteins. To test this hypothesis, we transiently expressed both Robo4 and Myc-tagged Slit2 in 293 cells, and examined their physical association in a Robo4 immunoprecipitation assay. We observed a physical association between Slit2 (c-Myc) and Robo4 in these cells (Figure 7B).

Since pretreatment with Slit2N inhibited c-Src signaling and there appeared to be a physical association between Slit2 and Robo4, we asked if the inhibition of c-Src signaling was a result of Slit2 sequestering Robo4 to deplete its cellular levels and render it unavailable for binding to a competing protein. To approximate this situation, we pretreated L-LECs with a mixture of Robo4 siRNAs or a control siRNA before incubating the cells with HIV-1 gp120. We did not observe the same inhibition of c-Src activation as we had with the Slit2N preincubation (Figure 7A). Instead, the constitutive activation of c-Src increased dramatically in the Robo4 knockdown cells as compared with the control siRNA-transfected cells (Figure 7C). These findings are consistent with the phenotype of Robo4 knockout mice which display heightened vascular permeability [17]. These data suggest that a sufficient endogenous level of Robo4 in lymphatic endothelium is necessary to block c-Src signaling, and that its binding to Slit2 is required to protect against lymphatic hyperpermeability. Additionally, HIV-1 gp120 did not enhance c-Src signaling in the Robo4 knockdown cells as it did in the control siRNA-transfected cells (Figure 7C). We hypothesize that the elevated constitutive level of c-Src kinase signaling in the Robo4 knockdown cells prevented HIV-1 gp120 from enhancing this effect in the L-LECs.

To determine if Src signaling is involved in HIV-1 gp120-induced lymphatic permeability, we pretreated L-LECs with a Src kinase inhibitor or a DMSO control before measuring HIV-1 gp120-induced permeability, as described previously. While treatment with HIV-1 gp120 resulted in increased permeability through the L-LEC monolayer preincubated with DMSO, HIV-1 gp120 had no effect on the L-LECs preincubated with a Src kinase inhibitor (Figure 7D). Taken together, we interpret these data to indicate that Src kinase signaling is required for HIV-1 gp120-induced lymphatic hyperpermeability, and that Slit2/Robo4 interactions can inhibit this signaling cascade.

### Robo4 Expression Levels Influence Lymphatic Hyperpermeability in L-LEC Monolayers

**Figure 8:**
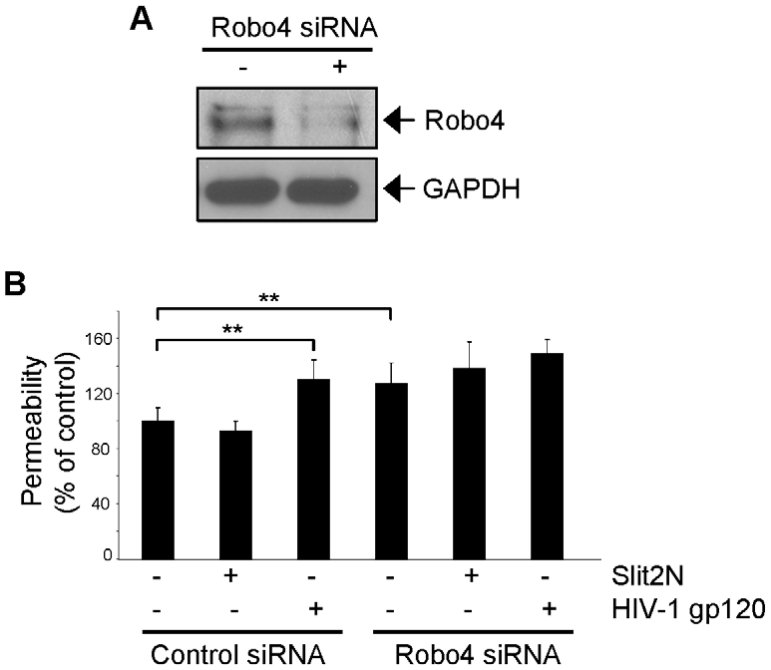
Robo4 expression levels influence HIV-1 gp120-induced lymphatic hyperpermeability. (**A**) Representative Western blot analysis of Robo4 expression in L-LECs, 24 hours after transfection with a mixture of Robo4-specific siRNAs or a negative control siRNA. GAPDH used as loading control. (**B**) Permeability through an L-LEC monolayer as previously described. L-LECs transfected with control siRNAs or Robo4-specific siRNAs were seeded into the upper chamber of transwell plates and incubated with Slit2N (500 ng/ml), HIV-1 gp120 (500 ng/ml), or a control, for 18 hours. Data indicate the mean ± SD of 3 independent experiments. (**p<0.01 for treatment with Slit2N or HIV-1 gp120 versus negative control of the L-LECs transfected with control siRNAs).

To characterize more precisely the role of Robo4 in HIV-1 gp120-induced effects on lymphatic permeability, we transfected L-LECs with control siRNAs or Robo4-specific siRNAs (to reduce Robo4 levels), and confirmed a decrease in Robo4 expression by Western blot analysis 24 hours later (Figure 8A). We compared the permeability of L-LEC monolayers expressing endogenous levels of Robo4 (Figure 8B, “Control siRNA” columns) and reduced levels of Robo4 (Figure 8B, “Robo4 siRNA” columns) in the presence or absence of Slit2N or HIV-1 gp120. In the L-LEC monolayers with endogenous levels of Robo4, incubation with Slit2N had no significant effect on permeability, but HIV-1 gp120 significantly increased the permeability of this monolayer. We observed a significantly higher basal level of permeability in the L-LEC monolayers with reduced Robo4 levels. Slit2N had no significant effect on the permeability of these monolayers, and HIV-1 gp120 failed to cause any significant change in the permeability of the L-LEC monolayers with reduced Robo4 levels. We hypothesize that HIV-1 gp120 did not enhance the permeability of these monolayers, because reducing Robo4 levels had already markedly increased their permeability. These data suggest that sufficient endogenous levels of Robo4 are required to maintain an intact lymphatic barrier.

### The Fibronectin Domains of Robo4 Are Critical for gp120-Induced Hyperpermeability of L-LEC Monolayers

**Figure 9:**
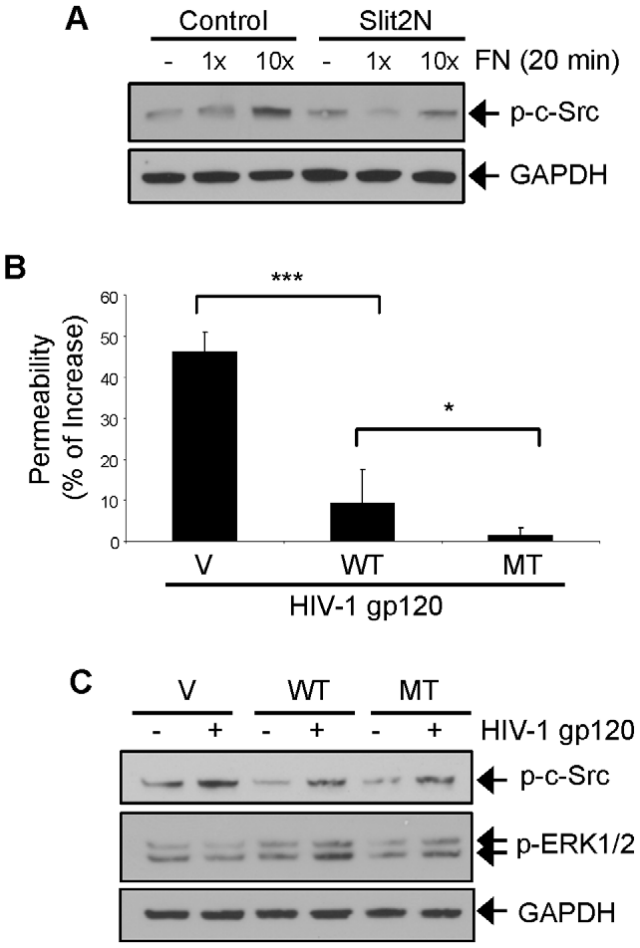
The fibronectin domains of Robo4 are critical for gp120-induced hyperpermeability of L-LEC monolayers. (**A**) Representative Western blot analysis of phosphorylated c-Src in L-LECs pretreated with Slit2N (500 ng/ml) or a control for 1 hour, then stimulated with fibronectin (FN) [1 µg/ml (1×); 10 µg/ml (10×)] for 20 minutes as indicated. GAPDH used as loading control. (**B**) Permeability through an L-LEC monolayer as previously described. L-LECs were transiently transfected with expression plasmids encoding wild-type Robo4 (WT), mutant Robo4 (MT), or a vector control (V). After 48 hours, cells were plated for the permeability assay per manufacturer's instructions. L-LEC monolayers were incubated overnight with 500 ng/ml HIV-1 gp120 or a control. Data are represented as the percentage increase in permeability of each cell type monolayer incubated with gp120 vs. control. Data indicate the mean ± SD of 3 independent experiments. (*p<0.05, ***p<0.001). (**C**) Representative Western blot analysis of phosphorylated c-Src and ERK1/2 in L-LECs transiently transfected with expression plasmids encoding wild-type Robo4 (WT), mutant Robo4 (MT), or a vector control (V). After 48 hours, the cells were serum-starved for 2 hours and stimulated with HIV-1gp120 (500 ng/ml) or a control for 15 minutes as indicated. GAPDH used as loading control.

The Robo4 receptor contains two fibronectin (FN) type III domains in its extracellular region [32], [33]. While a study by Kaur et al., demonstrated that these motifs are important for the interaction of Robo4 with fibronectin [38], no other function for the domains has been documented. Fibronectin regulates the permeability of vascular endothelium [44]. We observed that HIV-1 gp120 elevated FN levels and enhanced lymphatic monolayer permeability in L-LECs. Therefore, we examined the effects of fibronectin on c-Src activation, and its effects after pretreatment with Slit2N. We observed that fibronectin enhanced the activation of c-Src, and that pretreatment with Slit2N significantly inhibited the FN-induced activation of c-Src (Figure 9A). We hypothesize that Slit2N may be interacting with Robo4 to block the FN-induced c-Src activation, and that the FN domains of Robo4 may be involved in the inhibition of FN-induced c-Src activation by Slit2 and L-LEC monolayer hyperpermeability.

To explore the potential role of the Robo4 FN domains in HIV-1 gp120-induced effects, we compared the effects of HIV-1 gp120 on the permeability of L-LEC monolayers transfected with wild-type Robo4 (WT), mutant Robo4 (MT), which lacks the FN type III domains, or a vector control (V). We found that HIV-1 gp120 induced significantly less permeability in L-LEC monolayers with elevated levels of wild-type Robo4 as compared to those with endogenous Robo4 levels (Figure 9B). These results indicate that Robo4 inhibits HIV-1 gp120-induced permeability in L-LEC monolayers, and may protect the integrity of the lymphatic barrier after HIV-1 infection by interacting with Slit2. We also observed that HIV-1 gp120-induced permeability was inhibited to a significantly greater extent in the L-LEC monolayers transfected with mutant Robo4 vs. wild-type Robo4. In fact, treatment with HIV-1 gp120 resulted in no change in the permeability of the L-LEC monolayers expressing mutant Robo4 (Figure 9B). We interpret these results to indicate that the complexing of FN and Robo4 (through its FN type III domains) is necessary for HIV-1 gp120-induced hyperpermeability of L-LEC monolayers, and that the FN type III domains of Robo4 may be required for the interaction of HIV-1 gp120, Robo4 and FN.

To explore this hypothesis, we transiently transfected L-LECs with plasmids encoding wild-type Robo4 (WT), mutant Robo4 (MT), or a vector control (V). After 48 hours, we analyzed the effects of HIV-1 gp120 on c-Src pathway activation in each of the transfected cell types by Western blot analysis. We observed that the basal level of c-Src activation was lower in L-LECs with elevated Robo4 expression as compared to those with endogenous Robo4 expression (Figure 9C, WT/− and V/−, respectively). HIV-1 gp120 increased c-Src activation in both cell types (Figure 9C), however, overall HIV-1 gp120-induced c-Src activation levels were significantly lower in L-LECs with elevated Robo4 levels as compared to those with endogenous Robo4 levels (Figure 9C, WT/+ and V/+, respectively). In L-LECs expressing elevated levels of mutant Robo4 (MT), both basal c-Src activation and HIV-1 gp120-induced c-Src activation were equivalent to the L-LECs expressing elevated wild-type Robo4 (Figure 9C). These data indicate that elevated levels of Robo4 inhibit basal c-Src activation and HIV-1 gp120-induced c-Src activation. We hypothesize that since Slit2 inhibits c-Src activation, elevated Robo4 levels after transfection may magnify the effects of Slit2, by providing more receptors to which endogenous Slit2 can bind.

We also examined the levels of HIV-1 gp120-induced phosphorylation of ERK1/2, key signaling molecules for endothelial cell function, by Western blot analysis, using the same three groups of L-LEC transfectants. HIV-1 gp120 induced a significant increase in ERK1/2 phosphorylation in the L-LECs transfectants with elevated wild-type Robo4 expression as compared to those with endogenous Robo4 expression (Figure 9C). Although HIV-1 gp120 increased the phosphorylation of ERK1/2 in the L-LECs transfected with mutant Robo4, the increase was significantly lower than the wild-type Robo4 transfectants (Figure 9C). These data indicate that while the FN domains of Robo4 are not required for the inhibition of gp120-induced c-Src activation, they are required for gp120-induced phosphorylation of ERK1/2. We hypothesize that the FN domains of Robo4 may participate in the activation of other key signaling molecules like ERK1/2, however, further investigation is needed to fully understand their function.

## Discussion

The integrity of the lymphatic barrier requires a dynamic interaction between fibronectin, other extracellular matrix (ECM) proteins, and their receptors, cell-surface integrins [46], [53]. As a result of HIV-1-induced inflammation and increased protease expression, fibronectin fragments are detected in the blood of HIV-infected patients [54]–[56]. These fragments are believed to promote the transendothelial migration of HIV-1-infected and non-infected leukocytes, and to promote viral stability and cell-to-cell transmission [48], [56], [57]. We found that lymphatic endothelial cells produce elevated levels of cell-bound fibronectin after exposure to HIV-1 gp120 (Figure 2A). This elevation appeared to modulate the integrity of the lymphatic barrier. In particular, HIV-1 gp120 induced activation of α_5_β_1_ integrin which enhanced the physical complexing of HIV-1 gp120, fibronectin, α_5_β_1_ integrin and Robo4, and resulted in lymphatic hyperpermeability (Figures 1A, 2B, 2C, 4A and 4B).

While FN/integrin [45] and Slit2/Robo4 [17] interactions are both important for endothelial permeability, little is known about their relationship. α_5_β_1_ and α_v_β_3_ are two major integrins expressed on the surface of endothelial cells [45]. We and others have shown that integrin α_5_β_1_, but not α_v_β_3_, clustered in focal contacts of endothelial cells during stressful cellular conditions (Figure 4B) or incubation with fibronectin [58]–[60]. In this study, we found that Robo4 formed a complex with fibronectin and integrin α_5_β_1_ at low, basal levels in uninfected lymphatic endothelial cells (Figures 2B, 5A and 5B). Slit2N did not alter this association, which is important for maintaining the integrity of the lymphatic barrier (Figures 5A and 5C). However, exposure to HIV-1 gp120 enhanced the association of α_5_β_1_ and Robo4 (Figure 5A) and resulted in increased lymphatic permeability (Figures 1A and 5C). Moreover, pre-incubation with Slit2N blocked the HIV-1 gp120-induced enhanced complexing of Robo4 and α_5_β_1_, (Figure 5B) and lymphatic hyperpermeability was reduced (Figure 5C). These data suggest that α_5_β_1_ integrin may also participate in the effects of HIV-1 gp120 on lymphatic permeability, and Slit2 may help sustain the integrity of the lymphatic barrier after HIV-1 exposure.

Activation of the Src kinases modulates cytoskeletal remodeling and affects cell-to-cell and cell-to-ECM adhesion [61], [62]. Our data indicate that HIV-1 gp120 and Slit2 exert opposing effects on c-Src kinase signaling, namely, HIV-1 gp120 activates c-Src signaling, while pretreatment with Slit2N significantly reduces these effects (Figure 7A). Moreover, the enhancement or inhibition of Src kinase signaling and the resulting effect on lymphatic permeability is critically dependent on Robo4 levels (Figures 9A, 9B, and 9C).

Robo4 displays unique structure and function, but the relationship between these characteristics is largely unknown [26], [32], [33]. Although the first Ig domains of the Robos are highly conserved and important for Slit binding, direct binding of Slit2 to Robo4 is still debated [37]–[39]. The Robo proteins, including Robo4, contain fibronectin type III domains. Previous studies, which found that the FN domains were required for adhesion to fibronectin, suggest that these domains may play a central role in modulating vascular permeability [38]. Our data strongly support their function in Robo4-mediated lymphatic permeability upon HIV-1 gp120 stimulation, and imply a potential role for Robo4 in fibronectin-associated vasculopathies, such as HIV-associated pulmonary hypertension [63].

**Figure 10:**
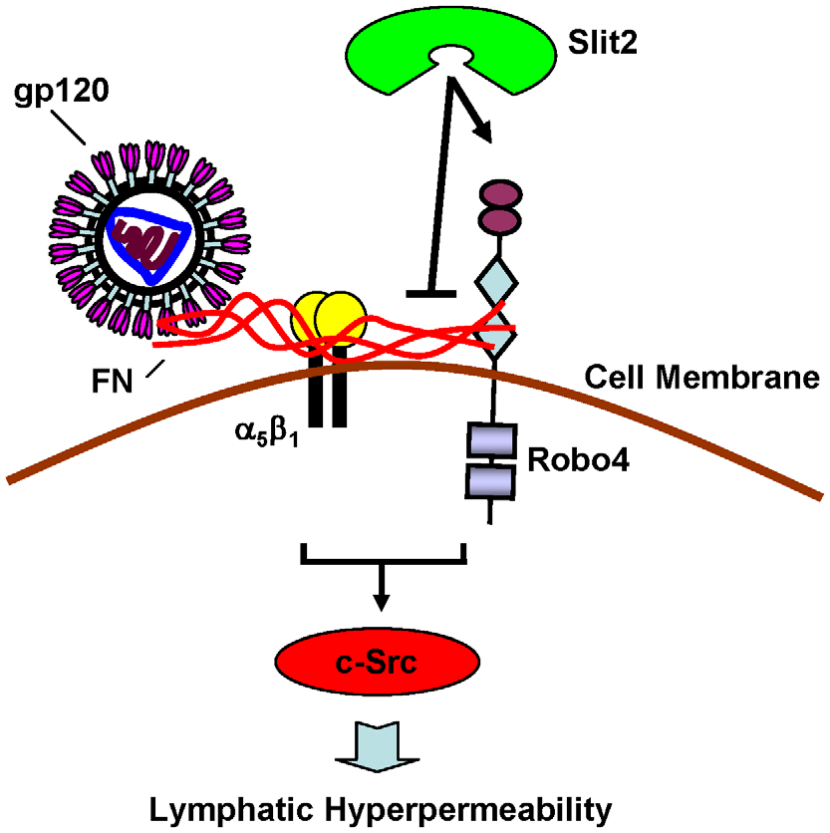
Hypothetical model for the interactions of HIV-1 gp120, FN, α_5_β_1_ integrin, Robo4 and Slit2, and their effect on the permeability of lymphatic endothelium. Robo4 and α_5_β_1_ integrin are expressed on the surface of lymphatic endothelial cells. Under physiologic conditions, endogenous Slit2 interacts with Robo4 via its Ig domains, and inhibits c-Src signaling. FN also interacts with Robo4 (via its FN type III domains), and binds to α_5_β_1_ integrin on the endothelial cell surface. This contributes further to the dynamic regulation of Robo4 signaling by Slit2 and FN, to maintain the integrity of the lymphatic endothelial barrier. Upon HIV infection, gp120 significantly elevates FN levels and complexes with FN. FN then activates α_5_β_1_ integrin, which results in enhanced intracellular signaling through α_5_β_1_ integrin, a stronger interaction between FN and Robo4, and disruption of Robo4 signaling. These changes activate the c-Src signaling pathway and induce hyperpermeability of the lymphatic endothelial barrier. Exogenous Slit2 may protect the lymphatic channels from HIV-induced vasculopathy by interacting with Robo4 to restore Robo4 signaling, block the c-Src pathway, and inhibit HIV-induced lymphatic hyperpermeability.

Based on our new data and that of others, we propose a hypothetical model for the interactions of HIV-1 gp120, FN, α_5_β_1_ integrin, Robo4 and Slit2, and their effect on lymphatic permeability (Figure 10). Robo4 and α_5_β_1_ integrin are transmembrane proteins expressed in lymphatic endothelium. We hypothesize that under normal, physiological conditions, soluble FN and Slit2 are expressed at low, basal levels, and they interact with Robo4 via its FN type III domains and Ig domains, respectively. FN also binds to α_5_β_1_ integrin, which is expressed on the endothelial cell surface. Under these conditions, the integrity of the lymphatic endothelial barrier is intact, and transmigration through the endothelial barrier is severely restricted. We propose that upon HIV infection, HIV-1 gp120 elevates FN levels significantly and complexes with FN. FN then activates α_5_β_1_ integrin, which results in enhanced intracellular signaling through α_5_β_1_ integrin, a significantly stronger interaction between FN and Robo4, and enhanced intracellular signaling through Robo4. These changes activate the c-Src signaling pathway and induce hyperpermeability of the lymphatic endothelial barrier. The resulting “leaky” barrier may facilitate the dissemination of HIV-1 and virus-infected cells throughout the body. Furthermore, we propose that elevated levels of Slit2 may protect the lymphatic channels from HIV-induced vasculopathy and HIV spread. We hypothesize that at sufficiently elevated levels, Slit2 will bind strongly to the Ig domains of Robo4 and inhibit c-Src pathway activation and HIV-1 gp120-induced lymphatic hyperpermeability. Slit2 may affect this inhibition by various means. A likely senario is that upon binding, Slit2 alters the protein conformation of Robo4, which may lessen/abolish its ability to interact with FN, alter α_5_β_1_ integrin intracellular signaling, and inhibit the activation of c-Src. In addition, the binding of Slit2 may alter also the signaling through Robo4, which may inhibit c-Src pathway activation and lymphatic hyperpermeability. Although our data strongly support this model, further investigation is needed to confirm it, or posit alternative mechanisms for the effects of HIV-1 gp120, FN, α_5_β_1_ integrin, Robo4 and Slit2 on lymphatic permeability.

Multiple studies indicate that the lymphatic channels play important roles in the establishment of HIV infection, and its dissemination throughout the host [1]–[4]. HIV-induced lymphadenopathy, including lymphoedema, is commonly seen among HIV-infected individuals with Kaposi's sarcoma, a vascular neoplasm which is derived from lymphatic endothelial cells [64], [65]; however, dysfunction of the lymphatic vasculature and its effects on HIV biology are largely unexplored. We established an *in vitro* endothelial monolayer model to study the effects of HIV on lymphatic permeability. In this model, HIV-1 gp120 and HIV-1 virions both induced lymphatic hyperpermeability, which was significantly inhibited by Slit2 preincubation (Figure 6B). These results suggest key roles for gp120, FN, and Slit2/Robo4 in HIV-associated lymphatic hyperpermeability, and implicate lymphatic hyperpermeability in HIV infection and spread throughout the body. Future studies to explore the traversion of HIV virions or virus-infected cells through the lymphatic endothelium and its contribution to HIV infection should provide more evidence on HIV-induced lymphatic hyperpermeability and HIV dissemination in a humanized mouse model of HIV infection.

In summary, we found that the balance between HIV-1 gp120/FN/α_5_β_1_ integrin-induced signaling and Slit2/Robo4-induced signaling in L-LECs modulates lymphatic monolayer permeability. Targeting these pathways may offer novel approaches to inhibit HIV-induced lymphatic injury, and limit the dissemination of HIV in the host.

## Materials and Methods

### Cells

Human embryonic kidney cells (293 cells) (Stratagene, La Jolla, CA, USA) were cultured in Dulbecco's modified Eagle's medium with 10% fetal calf serum. Primary human lung lymphatic endothelial cells (L-LECs) and dermal lymphatic endothelial cells (D-LECs) were purchased from Lonza, Inc. (Allendale, NJ, USA) and maintained in EBM-2 medium with EGM-2MV SingleQuots (Lonza, Inc.).

### Recombinant Proteins and HIV-1 Virions

Recombinant human Slit2N (the active fragment of Slit2) was provided by Dr. Dean Li, Department of Oncological Sciences at the University of Utah. The following reagents were obtained through the AIDS Research and Reference Reagent Program, Division of AIDS, NIAID, NIH: recombinant HIV-1_Ba-L_ gp120 (M-gp120) protein and HIV-1 virus (strain HIV-1 _Ba-L_, which was from Dr. Suzanne Gartner, Dr. Mikulas Popovic and Dr. Robert Gallo). Per NIH data sheet, HIV-1_Ba-L_ was originally isolated from a primary culture of adherent, human, infant lung tissue cells, and amplified in human monocytes/macrophages. Virus was harvested 10 days post-infection. This virus was used in the transendothelial monolayer permeability assays. HIV-1 gp120 LAV (III B) (T-gp120) was purchased from Protein Sciences Corporation (Meriden, CT, USA). HIV-1 gp120CM was purchased from ProSpec-Tany TechnoGene Ltd. (Ness Ziona, Israel). Src inhibitor-1 was purchased from Sigma-Aldrich, Corp. (St. Louis, MO, USA).

### Antibodies

Mouse monoclonal antibody to the HIV-1 gp120 was purchased from Advanced Biotechnologies Inc. (Columbia, MD, USA). Anti-integrin β_1_, anti-phospho-integrin β_1_ (Tyr783), and neutralizing anti-integrin α_5_β_1_ antibodies were purchased from Millipore Corp. (Billerica, MA, USA). Anti-phospho-Src kinase family antibodies were purchased from Cell Signaling Technology, Inc. (Beverly, MA, USA). All other antibodies were purchased from Santa Cruz Biotechnology, Inc. (Santa Cruz, CA, USA).

### RT-PCR

Total cell RNA was extracted using the RNeasy Mini Kit from Qiagen, Inc. (Valencia, CA, USA). RT-PCR was performed using a one-step RT-PCR kit from Clontech (Mountain View, CA, USA). Specific primers for CXCR4 and CCR5 were purchased from R & D Systems (Minneapolis, MN, USA). The primers for human Slit2 were synthesized by Invitrogen Corp. (Carlsbad, CA, USA). The sequences are: upstream: 5′-GGTGTCCTCTGTGATGAAGAG -3′; downstream: 5′- GTGTTTAGGACACACACCTCG -3′.

### Fluorescence Staining

Cells cultured in 8-well chamber slides (Thermo Fisher Scientific Inc., Waltham, MA, USA) were fixed with 4% (v/v) paraformaldehyde solution for 1 hour at room temperature, incubated in fresh permeabilization solution (0.1% sodium citrate in 1% Triton X-100 in 1× PBS) for 2 minutes on ice, incubated with 3% BSA/1× PBS on ice for 30 minutes, and then with anti-human CXCR4 rabbit polyclonal antibody or normal rabbit IgG (Millipore Corp.) at 4°C for 1 hour. The slides were then washed 3 times in 1× PBS, and incubated with a FITC-conjugated, goat anti-rabbit IgG antibody (Vector Laboratories, Burlington, CA, USA) at 4 °C for 30 minutes. The slides were washed again 3 times in 1× PBS, and then air dried and mounted with mounting medium (Vector Laboratories).

### Vascular Permeability Assay

Lymphatic endothelial cells were seeded in the top chamber of transwell plates, according to the manufacturer's instructions (Millipore Corp.), starved for one hour, and then incubated with different reagents or their respective controls as indicated. Subsequently, FITC-Dextran was added to the top chamber and allowed to permeate through the monolayer to the lower chamber for 5 minutes. The extent of permeability was determined by measuring the fluorescence of the solution in the lower chamber by a standard plate reader (BioTek Instruments, Inc., Vinooski, VT, USA). The gp120 control was prepared by boiling gp120 for 10 minutes to inactivate its protein activity while preserving its inherent endotoxin activity. It was employed here, and in all other experiments that required a gp120 control.

### Cell Stimulation, Immunoprecipitation, and Western Blotting

Cells were starved for 2 hours in serum-free media, and then stimulated as indicated. Cells were lysed in RIPA buffer (Cell Signaling Technology, Inc.) after stimulation. Immunoprecipitation and Western blotting were performed as described previously [66]. For quantitation, the ratio of protein expression, phosphorylation, or association vs total protein in each lane was obtained by densitometry with a gel imaging system (Cell Biosciences, Inc., Santa Clara, CA, USA).

### DNA Constructs

The pCMV6 Entry expression plasmid encoding Myc-DDK-tagged Slit2 was purchased from OriGene Technologies, Inc. (Rockville, MD, USA). The expression plasmid encoding RFP-tagged Robo4 was constructed as follows. Robo4 cDNA was amplified from the pCMV-SPORT6 containing Robo4 cDNA (Thermo Fisher Scientific Inc.), using primers purchased from Invitrogen Corp. (upstream sequence: 5′-GAGGCGATCGCATGGGCTCTGGAGACAGCCTCCTG-3′; downstream sequence: 5′-GCGACGCGTGGAGTAATCTACAGGAGAAGCACCAGC-3′). The purified PCR product was digested with *Sgf* I plus *Mlu* I, and inserted into the pCMV6-AC-RFP plasmid digested with same restriction endonucleases to create the pCMV6-AC-RFP-Robo4. To make the mutant Robo4 expression plasmid, we designed a pair of primers to amplify a section of the pCMV6-AC-RFP-Robo4 plasmid by PCR. The primers are: 5′-CCCCCCCCGCTAGCTCTAGGCTTGGGGCCCTCTGCAGGATC-3′ and 5′-TTTTTTTTGCTAGCCCTGTCTGCCTCCTTTTAGAGCAGGCC-3′. The PCR product was digested with Nhe1, purified, and ligated with T4 DNA ligase at 16°C overnight. The ligation product was used to transform competent DH5 α cells. Positive clones were screened and confirmed by DNA sequencing.

### siRNA Constructs

Specific Robo4 siRNAs and control siRNAs, purchased from Santa Cruz Biotechnology, Inc., were used to transfect L-LECs using HiPerFect transfection reagent from Qiagen, Inc.

### Plasmid DNA Transfection

Cells were grown to 60% confluence in tissue culture dishes. Transfections were done using Super Effectene transfection reagent according to the manufacturer's instructions (Qiagen, Inc.). At 3 hours post-transfection, cells were washed once with 1× PBS, then cultured in full medium for 48 hours. The transfection efficiency was determined by detection of red fluorescent cells under a fluorescent microscope (Nikon Diaphot 300, Tokyo, Japan).

### Confocal Microscopy

Cells cultured in 8-well chamber slides (Thermo Fisher Scientific Inc.) were serum starved for 2 hours, and then treated with HIV-1 gp120 as indicated. Subsequently, cells were fixed with 4% (v/v) paraformaldehyde for at least 1 hour at room temperature and permeabilized for 2 minutes on ice. Cells were then incubated with primary antibodies or normal IgG controls overnight at 4°C, and washed 3 times with 1× PBS. Fluorescence-conjugated secondary antibodies were added for 30 minutes at 4°C and the cells were washed 3 times in 1× PBS. Finally, the chambers were removed and coverslips were affixed with mounting medium containing DAPI (Vector Laboratories). Slides were examined under a Leica TCS-NT laser scanning confocal microscope (Leica Microsystems, Bannockburn, IL, USA).

### Data Analysis

Each experiment was repeated at least 3 times, and representative blots, images, or graphs are shown in the figures. Statistical significance was determined using the ANOVA test (*p<0.05).
