# Correction: Slit2/Robo4 Signaling Modulates HIV-1 gp120-Induced Lymphatic Hyperpermeability

**DOI:** 10.1371/annotation/5af1d62a-8262-4340-ad06-c450a50295e3

**Published:** 2012-07-26

**Authors:** Xuefeng Zhang, Jinlong Yu, Paula M. Kuzontkoski, Weiquan Zhu, Dean Y. Li, Jerome E. Groopman

There were errors in the Funding section. The correct funding information is as follows: "This work was supported by the National Institutes of Health (National Institute on Drug Abuse) grant 5DP1DA026197 and Dr. Dean Y. Li was funded by NHLBI. The funders had no role in study design, data collection and analysis, decision to publish, or preparation of the manuscript."

